# Limited Contribution of the Lactate Receptor HCAR1 to Exercise-Induced Behavioral and Hippocampal Adaptations

**DOI:** 10.1523/ENEURO.0005-26.2026

**Published:** 2026-07-23

**Authors:** Nikolaj Klahn, Vidar Jensen, Silje Bøyum, Cecilie Morland, Jens Randel Nyengaard, Mikkel Thy Thomsen

**Affiliations:** ^1^Core Center for Molecular Morphology, Department of Clinical Medicine, Aarhus University, Aarhus N 8200, Denmark; ^2^Letten Centre, Division of Anatomy, Department of Molecular Medicine, Institute of Basic Medical Sciences, University of Oslo, Oslo NO-0317, Norway; ^3^Department of Pharmacy, The Faculty of Mathematics and Natural Sciences, University of Oslo, Oslo NO-0316, Norway; ^4^Department of Pathology, Aarhus University Hospital, Aarhus N 8200, Denmark; ^5^Clinical Pharmacology, Pharmacy and Environmental Medicine, Department of Public Health, University of Southern Denmark, Odense M 5230, Denmark

**Keywords:** exercise, hippocampus, lactate signaling, major depressive disorder

## Abstract

Physical exercise influences hippocampal function and behavior, and lactate has emerged as a candidate signaling molecule linking metabolic activity to neuroplasticity. One proposed mediator is the hydroxycarboxylic acid receptor 1 (HCAR1), but its contribution to behavioral and hippocampal adaptations to exercise remains unclear. We combined studies of HCAR1 knock-out (KO) mice with analyses of human postmortem hippocampal tissue to assess whether HCAR1 is required for behavioral or synaptic responses to exercise and to characterize its spatial distribution in the human hippocampus. Wild-type and HCAR1 KO mice of either sex underwent a 3 week high-intensity interval treadmill program or sedentary handling. Behavioral responses were assessed using the splash test and three-chamber sociability assay, and dentate gyrus (DG) field recordings evaluated synaptic transmission and excitability. In parallel, HCAR1 expression was quantified in the hippocampal tissue from individuals with major depressive disorder (MDD) and nondepressed controls. Exercise reduced grooming and increased locomotion similarly across genotypes, indicating largely preserved behavioral responses in the absence of HCAR1. HCAR1 KO control mice exhibited delayed initiation of social interaction, not observed in exercised mice. Electrophysiology revealed subtle genotype-dependent differences in DG responsiveness following exercise, without major changes in short-term plasticity. In the small available cohort, HCAR1 showed a predominantly perivascular distribution across hippocampal subregions in both MDD and control cases. Together, these findings indicate that HCAR1 is not required for the primary behavioral and synaptic outcomes measured here following exercise, while leaving open a contribution to more specific aspects of hippocampal function under these or other conditions.

## Significance Statement

Exercise-induced increases in brain lactate have been proposed to influence hippocampal plasticity and behavior, in part through activation of the lactate receptor hydroxycarboxylic acid receptor 1 (HCAR1). By testing HCAR1 knock-out mice across behavioral and hippocampal electrophysiological outcomes and by characterizing HCAR1 distribution in the human hippocampal tissue, we find that loss of HCAR1 does not substantially alter the behavioral measures assessed or the main behavioral responses to exercise. Human hippocampal HCAR1 showed a predominantly perivascular distribution in both major depressive disorder cases and controls. These findings suggest that HCAR1 is not broadly required for the exercise-associated outcomes measured here while leaving open a role under specific physiological or pathological conditions.

## Introduction

Physical exercise produces broad effects on brain function, including changes in hippocampal plasticity, neurogenesis, vascular remodeling, and behavior (Van Praag et al., 1999; Schuch et al., 2016). These effects are relevant to several neuropsychiatric conditions, including major depressive disorder (MDD), where hippocampal structure and function are frequently altered and structured exercise can improve depressive symptoms in some patient populations (Czéh and Lucassen, 2007; Boldrini et al., 2012; Ross et al., 2023). However, the molecular mechanisms linking exercise to hippocampal adaptation and behavioral change remain incompletely understood.

A growing body of evidence implicates lactate as a signaling molecule linking metabolic activity to brain function. Exercise increases circulating lactate and, under high-intensity conditions, can elevate hippocampal lactate levels, where it has been linked to increased brain-derived neurotrophic factor (BDNF) expression, mitochondrial adaptations, and hippocampal plasticity (El Hayek et al., 2019; Müller et al., 2020; Hu et al., 2021; Hwang et al., 2023). In addition, peripheral or central lactate administration has been reported to influence neuronal activity, enhance stress resilience, and produce antidepressant-like behavioral effects in rodents (Carrard et al., 2018; Karnib et al., 2019; Hwang et al., 2023; Zhang et al., 2025). Together, these findings suggest that lactate signaling may contribute to exercise-associated adaptations in hippocampal function and behavior.

One proposed mediator of lactate signaling is the hydroxycarboxylic acid receptor 1 (HCAR1, also known as GPR81; HCA1), a G-protein–coupled receptor expressed in neural and vascular cell populations in the brain (Lauritzen et al., 2014; Morland et al., 2015; Hadzic et al., 2020). HCAR1 activation has been linked to modulation of neuronal excitability, angiogenic responses, and neurogenesis in selected brain regions (Lauritzen et al., 2014; Morland et al., 2017; de Castro Abrantes et al., 2019; Lambertus et al., 2021, 2024; Briquet et al., 2022; Kennedy et al., 2022). However, accumulating evidence indicates that the central effects of lactate are not exclusively mediated through HCAR1, as lactate can also act through metabolic, epigenetic, and redox-sensitive mechanisms (Carrard et al., 2018; Karnib et al., 2019). HCAR1 has therefore been proposed as a mediator of some—but not all—lactate-dependent effects of exercise. Consistent with this complexity, studies using genetic and pharmacological approaches have reported divergent behavioral outcomes following HCAR1 activation or loss of function, ranging from minimal behavioral effects to more pronounced alterations in social and exploratory behaviors (Morland et al., 2017; de Castro Abrantes et al., 2019; Nezhady et al., 2023).

Despite increasing interest in lactate–HCAR1 signaling as an exercise-associated pathway, it remains unresolved which behavioral and circuit-level adaptations associated with exercise are HCAR1-dependent and which may occur independently of lactate/HCAR1 signaling.

In the present study, we examined whether HCAR1 is necessary for behavioral and hippocampal adaptations to exercise. Specifically, we assessed behavioral responses and dentate gyrus (DG) synaptic function in wild-type (WT) and HCAR1 knock-out (KO) mice subjected to a 3 week treadmill intervention or sedentary conditions. In parallel, we evaluated the spatial distribution of HCAR1 in the postmortem human hippocampus from individuals with MDD and nondepressed controls, providing translational anatomical context and an exploratory assessment of whether marked differences in HCAR1 expression or perivascular distribution were apparent in the available cohort. By combining behavioral, electrophysiological, and anatomical analyses across species, this study assesses the contribution of HCAR1 to exercise-associated DG function and its distribution in the human hippocampus.

## Materials and Methods

### Animals

HCAR1 KO mice on a C57BL/6N background were generated by replacing the HCAR1 exon with a LacZ-neomycin cassette (Ahmed et al., 2010). WT C57BL/6N littermates served as controls. Mice were housed in Green Line Sealsafe Plus GM500 cages (Tecniplast) in same-sex groups under a 12 h light/12 h dark cycle with *ad libitum* access to food and water. Environmental enrichment included bedding, nesting material, and semiopaque shelters. Cages were changed biweekly. At 5 weeks of age, mice were ear-tagged for identification and genotyping. Genomic DNA was extracted (either GenElute, Sigma-Aldrich, or QIAamp, Qiagen), and PCR was performed using primers targeting HCAR1 and LacZ; products were run on agarose gels to confirm homozygous genotypes. Welfare was assessed daily, and predefined euthanasia criteria based on humane endpoints were applied throughout the study. The mice were weighed at least weekly during the intervention period. All protocols were approved by the national and local authorities (FOTS 28420), and personnel were FELASA certified.

### Intervention

**Figure 1.**
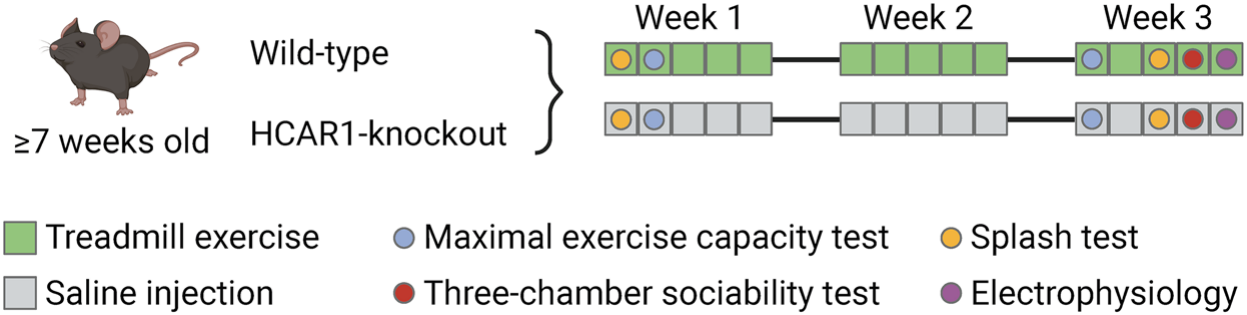
Experimental timeline and behavioral assessment overview for HCAR1 KO and WT mice. The figure was prepared with BioRender.

At ≥7 weeks of age, HCAR1 KO and WT mice were randomly allocated to exercise or control groups (*n* = 7–11 per group) while ensuring equal sex ratio across groups (21 males and 15 females mice in total). Exercising mice underwent treadmill running 5 d/week (Exer-3/6 treadmill, Columbus Instruments) on a 25° incline (Fig. 1). Each session included a 10 min warm-up at 5 m/min followed by 10 × 4 min intervals at 75% of maximal exercise capacity test speed (assessed on Days 2 and 15), interspersed with 2 min active recovery at 5 m/min. Control mice were handled identically but remained sedentary and received intraperitoneal isotonic saline injections (20 ml/kg). A planned lactate-injection group was excluded from the final analyses because the injection protocol failed as a valid lactate intervention. Specifically, a preparation error resulting in a hyperosmotic injection solution resulted in stress responses unrelated to the intended treatment, as indicated by overt behavioral signs of distress and markedly elevated corticosterone levels relative to the other groups. Because these animals could therefore not be interpreted as a pharmacological lactate-stimulation group, they were excluded from hypothesis testing and are not included in the main analyses.

### Behavioral assessment

All behavioral assays were performed ≥4 h after the end of the daily exercise session.

#### Splash test

On Days 1 and 17 (Fig. 1), mice were subjected to a splash test, which is widely used as an index of self-care and motivational behavior. The mice were placed in a 41 × 41 cm plexiglass chamber for a 10 min habituation. Two sprays (0.13 ml each) of 100 mg/ml sucrose solution were applied to the dorsal coat from ∼10 cm distance. Behavior was recorded for 5 min via overhead camera. ANY-maze (v. 7.13) tracked locomotion; grooming (forepaw contact with sucrose-coated fur) was scored manually. Chambers were cleaned with detergent and dried between trials.

#### Three-chamber sociability test

On Day 18 (Fig. 1), a three-chamber sociability test was performed. The apparatus consisted of a clear plexiglass arena (41 × 62 cm) with a central chamber and two side chambers (10 × 10 cm openings). Each side chamber contained a wire cup on a raised platform. Mice were habituated for 10 min with all chambers accessible and then confined to the center chamber while an age- and sex-matched unfamiliar mouse was placed under one of the cups. The other cup remained empty. Barriers were removed, and exploration was recorded for 10 min. ANY-maze quantified investigative behavior (time within 3 cm of and oriented toward each cup), latency to first social approach, total distance traveled, and number of center entries. Sociability index was calculated as time investigating the novel mouse/total investigation time. All items were cleaned and allowed to dry between trials.

### Electrophysiology

#### Hippocampal slice electrophysiology

On Day 19 (Fig. 1), mice were anesthetized with isoflurane and decapitated. Brains were rapidly transferred to ice-cold carbogenated dissection artificial cerebrospinal fluid (aCSF) containing the following (in mM): 124.08 NaCl, 2.01 KCl, 1.25 KH_2_PO_4_, 3.98 MgSO_4_, 26.01 NaHCO_3_, 1.0 CaCl_2_, and 12.21 d-glucose. The cerebellum and frontal cortices were removed, and ventral hippocampi were isolated and mounted dorsal side down on a vibratome (Leica VT1200). Transverse slices (400 µm) were cut at room temperature and incubated for 1 h at 32°C in holding aCSF (identical to dissection aCSF but supplemented with 4 mM CaCl_2_), continuously bubbled with 95% O_2_/5% CO_2_, and were kept here until recording (<5 h).

#### Recording conditions

Slices were transferred to a recording chamber kept at 31°C, which was perfused with holding aCSF containing 10 µM picrotoxin. Glass electrodes filled with holding aCSF were placed in the molecular layer of the DG, targeting the medial perforant path. Field excitatory postsynaptic potentials (fEPSPs) were recorded at a sampling rate of 20 kHz with a 3 kHz low-pass filter and monitored using the Clampex software (v. 10.7.0.3).

#### Electrophysiological protocols

To assess synaptic transmission, a paired-pulse protocol (50 ms interstimulus interval, six pairs at 0.1 Hz) was applied. The stimulation intensity was adjusted to produce (1) recordings with a presynaptic volley of 0.5 mV amplitude to quantify synaptic transmission, (2) recordings with a stimulus intensity below the threshold needed for the second pulse to elicit a population spike to quantify paired-pulse depression, and (3) recordings with a near-threshold stimulus intensity for the first pulse to elicit a population spike to quantify the population spike threshold.

#### Electrophysiological data processing

A custom MATLAB script was used to analyze the electrophysiological data and to calculate an average value from each slice. Traces with low amplitude or high instability were excluded from analysis.

### Corticosterone measurement

Immediately before dissection for electrophysiology, in the first half of the afternoon (∼12:00–15:00), trunk blood was collected, and serum was sampled by allowing the samples to coagulate at room temperature, followed by centrifugation at 2,290 × *g* for 15 min at 4°C (Universal 320R Centrifuge, Hettich) and stored at −20°C until corticosterone measurements were performed. The amount of corticosterone in mouse serum was measured using a competitive ELISA Kit (catalog #EIACORT). Samples were diluted 1:100 and run in duplicate as recommended by the manufacturer. The absorbance was read at 450 nm (Wallac Victor Nivo, Perkin Elmer).

### Human hippocampal immunohistochemistry

Formalin-fixed paraffin-embedded hippocampal tissue from a large, national brain biobank was used with approval from the national ethical research committee (case number, 2308632; doc. number, 2858446). For this study, samples were selected from individuals diagnosed with depression (ICD-8 codes equivalent to ICD-10 F32/F32.8) or without psychiatric diagnoses. The control tissue was obtained from individuals with no recorded MDD diagnosis who died from causes unrelated to neurological or psychiatric disease.

#### Tissue preparation and immunohistochemical staining

Two to three 2-µm-thick sequential sections were cut (LEICA RM2255) from the paraffin-embedded hippocampal human tissue and mounted on SuperFrost Plus slides (Thermo Fisher Scientific) and then stored at 4°C. Sections were deparaffinized at 60°C (30 min), washed twice in Histoclear (15 min), rehydrated through graded ethanol, and subjected to heat-induced epitope retrieval in Tris-EDTA, pH 9.0, by microwave (5 min + 5 × 1 min).

Sections were circumscribed with a hydrophobic PAP pen and permeabilized in 0.3% Triton X-100 in Tris-buffered saline (TBS), pH 7.4 (3 × 10 min). Autofluorescence was quenched using TrueBlack (Biotium), followed by TBS rinses. Blocking was performed in 1% skim milk in TBS (3 × 20 min). Primary antibodies were applied overnight in blocking buffer, rabbit anti-HCAR1 (2 μg/ml, PA5-114741, Thermo Fisher Scientific) and mouse anti-COL4 (0.4 μg/ml, ab273607, Abcam), or isotype controls, rabbit IgG (2 μg/ml, AB_2532938, Thermo Fisher Scientific) or mouse IgG (0.4 μg/ml, ab37355, Abcam).

Following rinses, sections were incubated with Alexa Fluor 555 goat anti-rabbit IgG (AB_2535849, Thermo Fisher Scientific) and Alexa Fluor 488 goat anti-mouse IgG (A11001, Thermo Fisher Scientific) for 60 min. DAPI (0.1 μg/ml) was applied for nuclear counterstaining, and slides were mounted with Fluoromount (F4680, Sigma-Aldrich), sealed with nail polish, and stored at 4°C. For anatomical reference, one section per subject was stained with toluidine blue.

Slides were imaged on a Leica DM6000 B epifluorescence microscope (Leica Microsystems) equipped with a Ludl motorized *x*–*y* specimen stage (99S121, LUDL Electronic Products) and an Olympus DP72 digital color camera (12.8 megapixel, Olympus Denmark) using the Leica HCCX FL Plan 10× objective (NA 0.25) and analyzed with the Visiopharm software (version 2020.08). Hippocampal subregions were defined based on Chen et al. (2020). The exposure time for each wavelength and other microscope settings were optimized on pilot slides before obtaining the final images and were then kept constant throughout the image collection. Using systematic uniform random sampling, ∼15 fields of view per region per section were selected. Manual focus was optimized for each field of view.

### Data analysis, statistics, and visualization

Behavioral data (grooming time, latency, locomotion, sociability metrics), electrophysiological outcomes, and corticosterone measurements were analyzed using two-way ANOVA (genotype × treatment) followed by Tukey-adjusted pairwise comparisons (intergenotype and intertreatment) to adjust for multiple comparisons (R v. 4.5.0, “emmeans” v. 1.11.1). Pre–post differences in splash test behavior were evaluated using paired *t* tests with Benjamini–Hochberg *p* value correction. Human immunohistochemistry data (HCAR1 intensity per nucleus, capillary area, HCAR1/capillary ratio) were obtained through a custom MATLAB script, which filtered out noise, counted nuclei using a watershed function on the DAPI channel (blue), identified and masked capillary area from the Col IV channel (green), and calculated the average intensity of the HCAR1 channel (red) at increasing distances from the capillaries. The output data were analyzed using a two-way ANOVA (diagnosis × region), followed by post hoc Tukey tests. All data were assessed for normality and homoscedasticity using fitted versus residual plots, *Q*–*Q* plots, and density plots. Significance was defined as *p* < 0.05. All plots except for microscopy images were prepared using the R package “ggplot2” v 4.0.0, and for boxplots, lines indicate the median, boxes the 25–75% percentiles range, and whiskers the data range within 1.5 times the interquartile range in either direction (standard settings for “ggplot2’s “geom_boxplot” function). All data are presented as mean ± SD unless otherwise stated.

## Results

### Exercise reduces grooming and increases locomotor activity in the splash test

**Figure 2.**
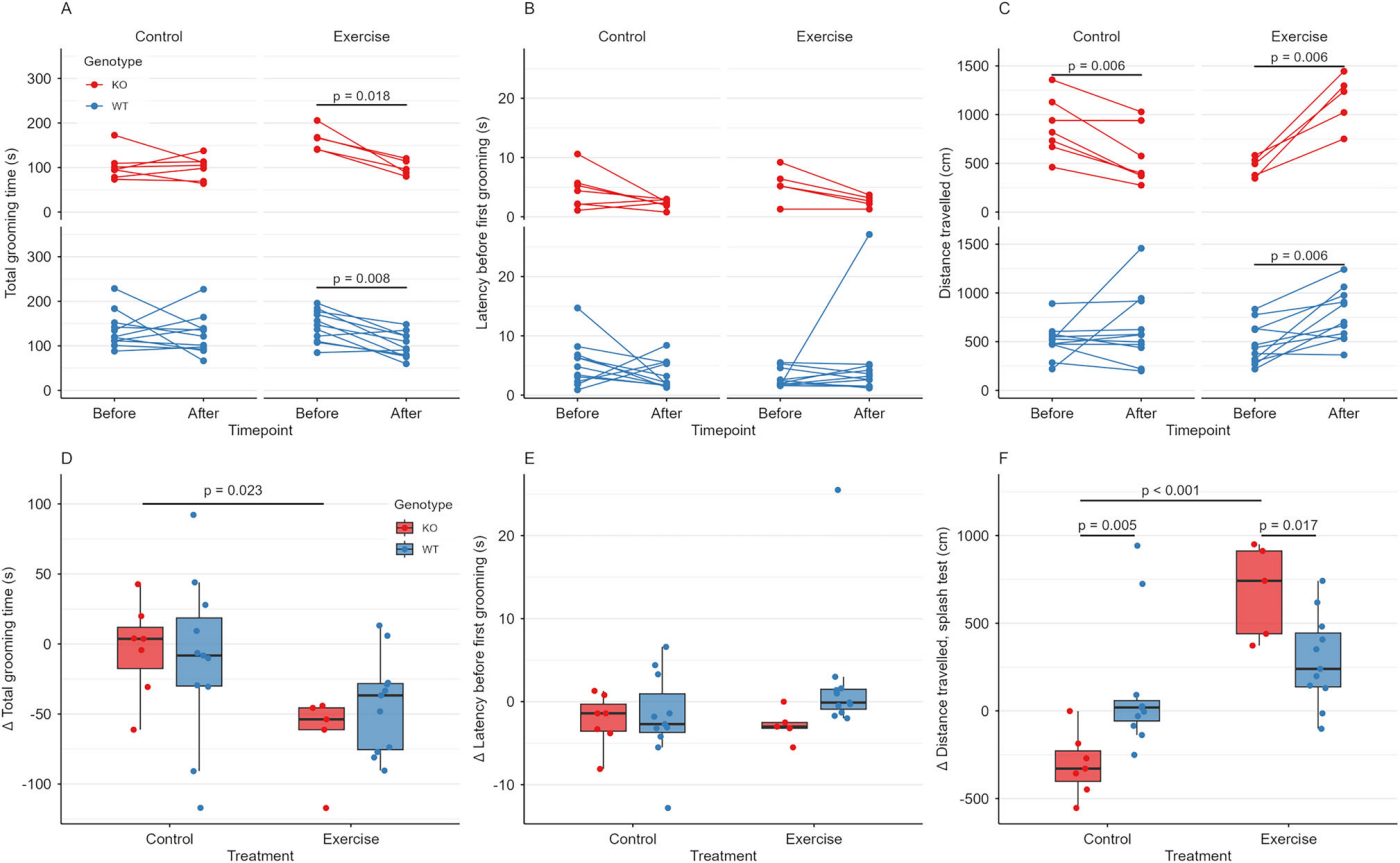
Splash test performance before and after exercise in WT and HCAR1 KO mice. ***A*–*C***, The measurement before and after the 3 week treadmill exercise intervention for each subject, subdivided by treatment and genotype. ***A***, Total grooming time. ***B***, Latency to first grooming event. ***C***, Total distance traveled during the splash test. ***D*–*F***, The corresponding change scores (post minus pre). ***D***, Difference in total grooming time. ***E***, Difference in latency to first grooming. ***F***, Difference in distance traveled. For all plots, blue represents WT mice; red represents HCAR1 KO mice. Statistically significant differences are indicated. Paired *t* tests were used for panels ***A–C*** and two-way ANOVAs for panels ***D–F***. *P* values were adjusted for multiple comparisons. WT, *n* = 11 in each group; HCAR1 KO, *n* = 5 in the exercise group; *n* = 7 in the control group.

After the 3 week treadmill intervention, grooming time in the splash test was significantly reduced in both WT and HCAR1 KO mice, whereas grooming times in sedentary controls of both genotypes remained largely unchanged (Fig. 2*A*,*D*). In parallel, exercise increased the distance traveled during the test across genotypes, consistent with a robust exercise effect on locomotor activity (Fig. 2*C,F*). These effects indicate that both genotypes exhibited consistent changes in splash test behavior following the treadmill intervention, without a significant genotype × treatment interaction for these measures. Baseline differences between genotypes were observed in sedentary animals: HCAR1 KO control mice showed lower baseline grooming times (Fig. 2*A*, 104 ± 33 s relative to a grand mean baseline of 136 ± 39 s) and higher baseline locomotor activity (Fig. 2*C*, 873 ± 300 cm relative to a grand mean of 568 ± 255 cm), which may have reduced the dynamic range of this behavioral test in this group.

### Subtle genotype- and exercise-dependent differences in sociability behavior

**Figure 3.**
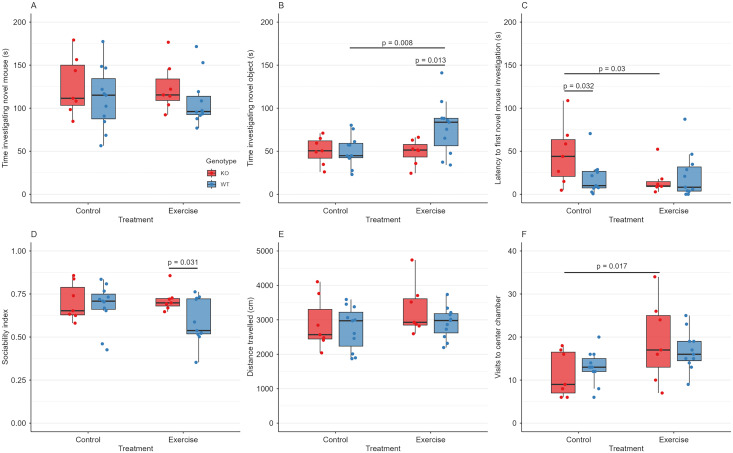
Three-chamber sociability test outcomes following exercise in WT and HCAR1 KO mice. ***A***, Time spent investigating the novel mouse. ***B***, Time spent investigating the novel object. ***C***, Latency to initiate interaction with the novel mouse. ***D***, Sociability index. ***E***, Total distance traveled during the test. ***F***, Number of visits to the center chamber. For all plots, blue represents WT mice; red represents HCAR1 KO mice. Statistically significant differences are indicated. Two-way ANOVAs with *p* value adjustment for multiple comparisons were used. WT, *n* = 11 in each group; HCAR1 KO, *n* = 7 in each group.

In the three-chamber sociability test, total distance traveled did not differ between groups (Fig. 3*E*), indicating that locomotor activity did not confound interpretation of sociability measures in this assay. Behavioral differences between groups were modest: exercised WT mice displayed a slightly lower sociability index driven by increased investigation of the novel object (Fig. 3*A,B,D*), while HCAR1 KO exercise mice showed an increased number of center-chamber visits relative to KO controls (Fig. 3*F*). Additionally, we observed a prolonged latency to initiate interaction with the novel mouse in HCAR1 KO control mice, indicating a subtle alteration in social approach (Fig. 3*C*), which was not observed in exercised HCAR1 KO mice.

### Genotype-dependent differences in exercise-associated DG excitability

**Figure 4.**
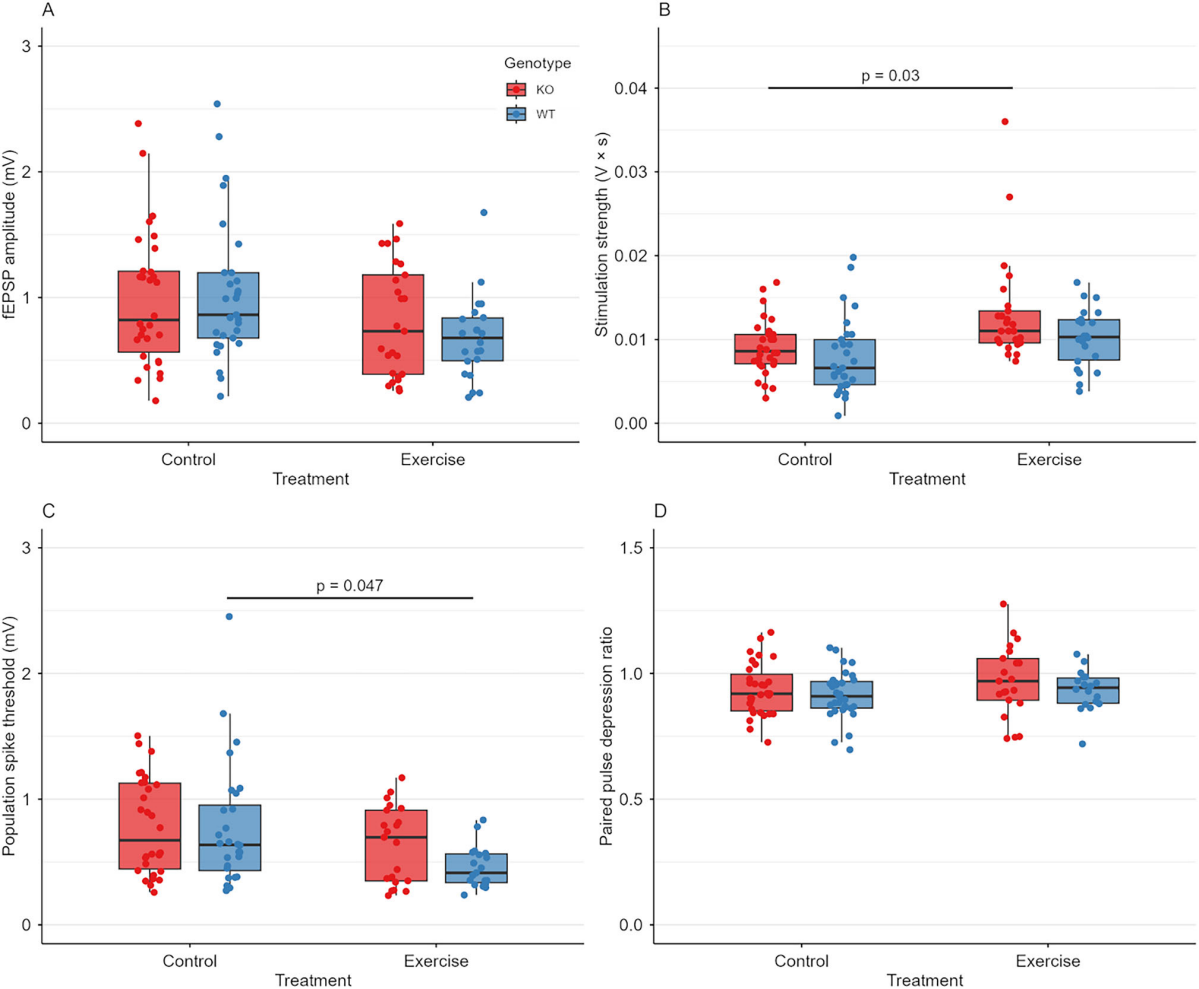
Electrophysiological effects of exercise and HCAR1 KO in mouse hippocampal slices. Field recordings from hippocampal slices stimulated with a paired-pulse protocol (50 ms interstimulus interval, six pairs at 0.1 Hz) were used to assess synaptic and neuronal excitability following exercise treatment in WT and HCAR1 KO mice. ***A***, The amplitude of the fEPSP elicited by a 0.5 mV prevolley. ***B***, The stimulation strength needed to elicit a 0.5 mV prevolley amplitude. ***C***, The fEPSP amplitude at the population spike threshold. ***D***, Ratios of the fEPSP amplitudes between the second and the first pulse. For all plots, blue represents WT mice, red represents HCAR1 KO mice, and each point represents the mean of a slice. Statistically significant differences are indicated. Two-way ANOVAs with *p* value adjustment for multiple comparisons were used. *n* = 7 mice per group, except for WT exercise, where *n* = 6.

In the DG, exercise produced subtle changes in synaptic responsiveness. In HCAR1 KO mice, the stimulation intensity required to evoke a 0.5 mV presynaptic volley was slightly higher after exercise than in sedentary controls (Fig. 4*B*), suggesting slightly reduced synaptic responsiveness. In contrast, exercised WT mice showed a significantly lower fEPSP amplitude at the population spike threshold compared with WT controls (Fig. 4*C*), indicating altered coupling between synaptic input and population spiking. Paired-pulse depression did not differ between groups, arguing against major changes in short-term synaptic plasticity (Fig. 4*D*).

#### Endpoint corticosterone levels reveal genotype-dependent differences after intervention

**Figure 5.**
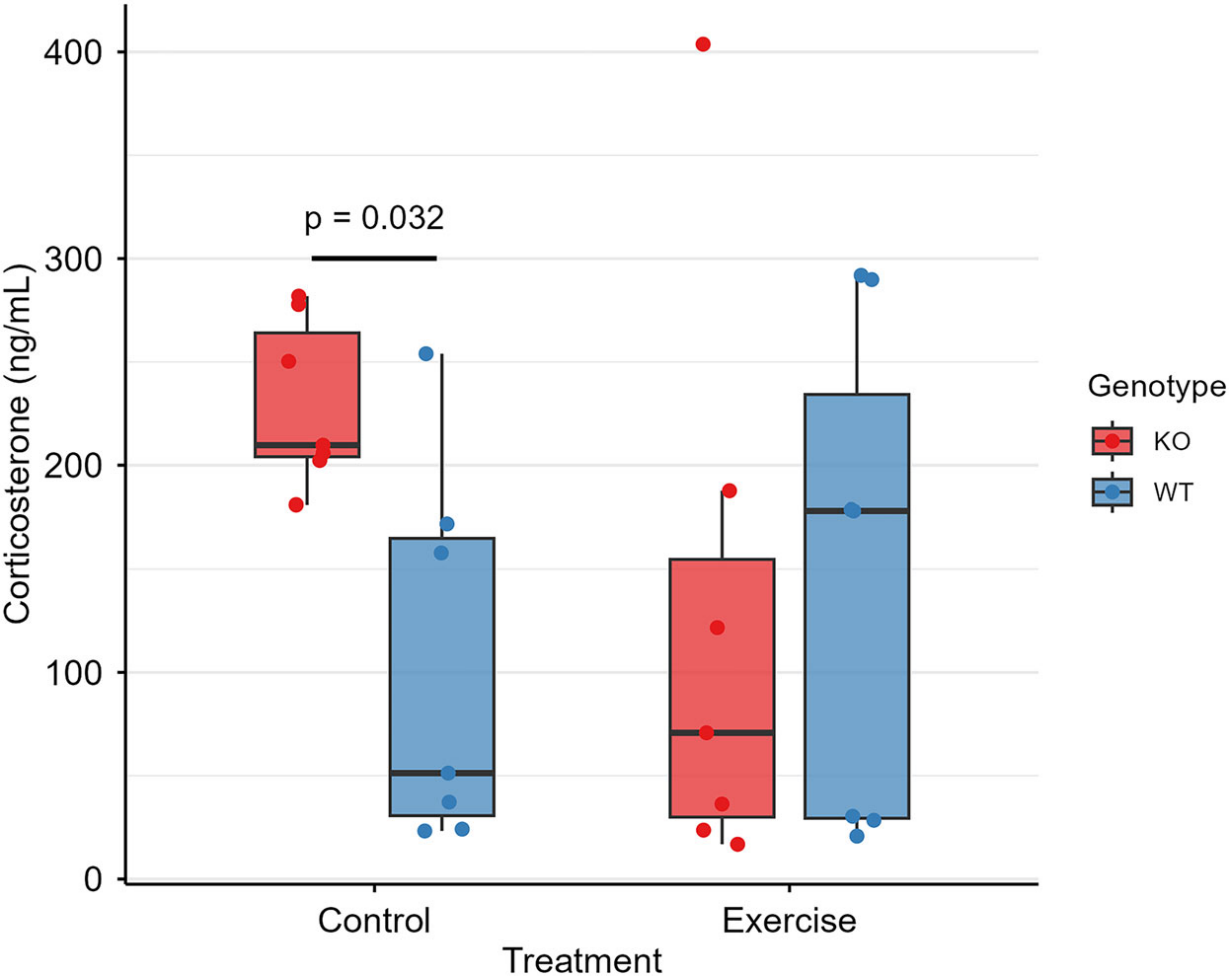
Corticosterone levels in WT and HCAR1 KO mouse serum following intervention. Serum corticosterone concentrations were measured at the study endpoint in WT and HCAR1 KO mice subjected to either a 3 week treadmill intervention or sedentary controls. Blue represents WT mice; red represents HCAR1 KO mice. Two-way ANOVA with *p* value adjustment for multiple comparisons were used; significant difference is indicated in the figure. *n* = 7 for all groups.

To assess whether the exercise paradigm induced a sustained stress response at the time of sacrifice, corticosterone was measured in serum collected ≥4 h after the final treatment session, during the first half of the afternoon. Corticosterone levels were variable across groups, and no significant increase was observed in exercised WT mice compared with sedentary controls (Fig. 5).

In contrast, sedentary HCAR1 KO mice displayed consistently elevated corticosterone levels relative to sedentary WT mice (*p* = 0.032). Exercised HCAR1 KO mice exhibited lower and more heterogeneous corticosterone levels, overlapping with those observed in WT groups. These findings indicate that corticosterone levels by the end of the treatment regimen do not reflect a uniform exercise-induced elevation but instead suggest genotype-dependent differences in baseline or recovery-phase corticosterone levels.

### HCAR1 expression in the human hippocampus shows conserved perivascular enrichment

**Figure 6.**
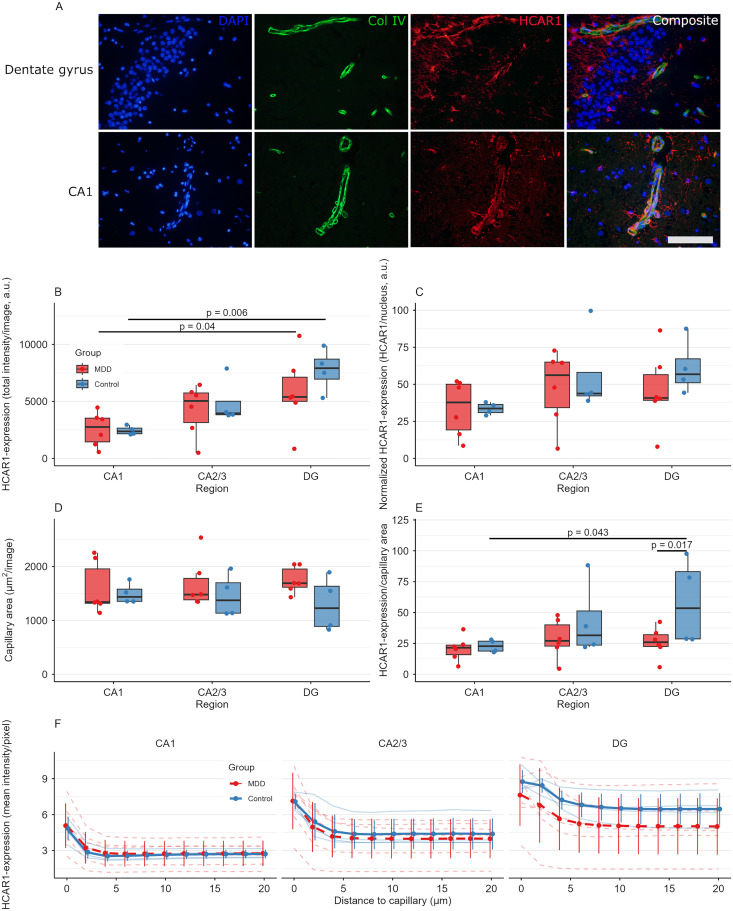
HCAR1 expression and capillary association in the hippocampal brain tissue from MDD cases and controls. Immunofluorescence (IF) staining of postmortem hippocampal sections from individuals with MDD and nondepressed controls was used to assess HCAR1 expression and its spatial association with capillaries. ***A***, Representative images from the DG and CA1, stained for nuclei (DAPI), capillaries (collagen IV), and HCAR1, and a composite image. Scale bar, 100 µm. ***B***, HCAR1 expression quantified as mean fluorescence intensity per image per region across CA1, CA2/3, and DG. ***C***, HCAR1 expression adjusted by cell number profiles (by nuclei stain). ***D***, Capillary area by collagen IV-positive pixels. ***E***, The ratio of HCAR1 expression to capillary area. ***F***, HCAR1 expression (fluorescent intensity/pixel) relative to the nearest capillary (collagen IV-positive pixels). For panel ***F***, semitransparent lines show the individual hippocampus results, and the bold lines indicate the group means. Solid lines represent control hippocampi and dashed lines represent MDD. For all panels, blue represents control hippocampi, and red represents MDD hippocampi. Statistically significant differences are indicated. Two-way ANOVAs with *p* value adjustment for multiple comparisons were used. MDD, *n* = 6; control, *n* = 4; results are exploratory.

Quantitative immunofluorescence analysis demonstrated that HCAR1 expression was enriched in perivascular regions of the human hippocampus, with fluorescence intensity decreasing as a function of distance from collagen IV-positive capillaries (Fig. 6*A,F*). This pattern was observed across hippocampal subregions. In exploratory comparisons between MDD and control cases, mean HCAR1 intensity and capillary area did not differ significantly across CA1, CA2/3, and DG, although trends toward lower HCAR1 signal and higher capillary area were observed in the DG of MDD cases (Fig. 6*C*,*D*). Regional differences in HCAR1 expression were present (Fig. 6*B*) but were attenuated after adjustment for cell number (Fig. 6*C*). Analysis of HCAR1-to-capillary ratios revealed regional variation (Fig. 6*E*), suggesting that HCAR1 expression is not solely determined by local vascular density.

## Discussion

In this cross-species study, we examined whether the lactate receptor HCAR1 contributes to behavioral and hippocampal adaptations associated with exercise. Overall, our findings indicate a limited contribution of HCAR1 to the outcomes assessed here. HCAR1 deletion did not produce a robust behavioral phenotype, and the 3 week treadmill intervention elicited comparable changes in splash test performance across genotypes. Subtle genotype-dependent differences were observed in social approach behavior and DG excitability, suggesting that HCAR1 may modulate specific aspects of hippocampal circuit function without driving large-scale behavioral outcomes. In parallel, analysis of human hippocampus revealed a conserved perivascular distribution of HCAR1, with no apparent alteration of hippocampal expression or perivascular distribution in the available small MDD cohort. Together, these findings do not support a broad requirement for HCAR1 for the baseline behavioral measures assessed here or for the primary adaptations to exercise observed in this paradigm while remaining consistent with more subtle, context-dependent effects at the circuit level.

Interpreting behavioral outcomes in rodents remains challenging, as assays such as the splash test and three-chamber sociability task provide indirect measures of affective and social function (Pollak et al., 2010). In our study, genotype-related effects were modest and primarily restricted to social behavior, where HCAR1 KO control mice exhibited a prolonged latency to initiate interaction with a novel conspecific, a phenotype not observed following exercise. Previous studies have reported divergent behavioral findings in HCAR1 KO mice, ranging from minimal changes (Morland et al., 2017; de Castro Abrantes et al., 2019) to more pronounced autism-like phenotypes (Nezhady et al., 2023). In parallel, lactate has been reported to influence behavior through multiple pathways that are not exclusively HCAR1-dependent, suggesting that the behavioral findings in the present study may reflect receptor-independent lactate actions or HCAR1-independent compensatory mechanisms (Carrard et al., 2018; Karnib et al., 2019). These observations suggest that HCAR1 deletion does not consistently produce a strong behavioral phenotype under baseline conditions and that lactate-related behavioral effects may not depend exclusively on HCAR1-mediated signaling.

Exercise induced clear behavioral changes in the splash test, including reduced grooming and increased locomotor activity in both genotypes. While such changes are often interpreted in the context of affective behavior, forced treadmill paradigms have been proposed to introduce a stress component (Fuss et al., 2010). Corticosterone measurements provided additional context. Samples were collected ∼4 h after any intervention, during the early afternoon, within a common collection window intended to reduce systematic circadian variation. Because samples were collected after this recovery interval, these values likely reflect a postexercise recovery state rather than the acute hormonal response to the exercise session. Under these conditions, we did not observe a consistent elevation of corticosterone in exercised animals. Instead, sedentary HCAR1 KO mice displayed elevated and less variable corticosterone levels compared with WT controls, suggesting genotype-dependent differences in baseline or recovery-phase corticosterone levels, the mechanistic basis of which was not investigated in the present study. This may contribute to the subtle baseline differences observed in social behavior. Importantly, these data do not support a uniform, sustained stress response induced by the exercise protocol. More broadly, this interpretation aligns with evidence that lactate promotes stress resilience primarily under more demanding stress paradigms, indicating that its functional relevance is context-dependent (Karnib et al., 2019).

Electrophysiological analyses revealed modest, genotype-dependent differences in DG responsiveness following exercise. Exercised WT mice exhibited reduced fEPSP amplitude at the population spike threshold, an effect absent in HCAR1 KO animals, consistent with a subtle influence of HCAR1 on input–output coupling. These observations reveal subtle, genotype-dependent differences in how exercise affects DG excitability, though the underlying mechanisms and the specific contribution of HCAR1 loss versus compensatory adaptations cannot be determined from the present data. Previous studies indicate that hippocampal neurogenesis is not strongly altered by HCAR1 deletion (Lambertus et al., 2021; Hwang et al., 2023), suggesting that neurogenic capacity is unlikely to fully explain the effects observed here. This is consistent with evidence that lactate influences hippocampal plasticity through multiple pathways, including BDNF-related and SIRT1-dependent mechanisms, which are not necessarily HCAR1-dependent (El Hayek et al., 2019; Carrard et al., 2021; Hwang et al., 2023). In addition, HCAR1 signaling depends on ligand availability and may primarily influence neuronal activity under conditions of elevated lactate (de Castro Abrantes et al., 2019; Herrera-López et al., 2020; Briquet et al., 2022), which may explain the limited baseline differences observed between genotypes.

Our analysis of human hippocampal tissue provides translational anatomical context for these findings. HCAR1 exhibited a consistent perivascular enrichment across hippocampal subregions, consistent with previous reports linking HCAR1 to vascular-associated lactate signaling and angiogenic responses in mice (Morland et al., 2017). In exploratory comparisons between MDD and control cases, neither overall HCAR1 expression nor capillary-associated signal differed significantly. Because the cohort was limited in size and the analysis was not cell type-specific, these data cannot exclude subtle diagnosis-related alterations. Within these limitations, however, we did not observe evidence for marked alterations in hippocampal HCAR1 expression or perivascular distribution in MDD. Thus, the human data are best interpreted as translational anatomical context for HCAR1 distribution in the human hippocampus, while the small cohort available here was insufficient to assess diagnosis-related differences in HCAR1 expression or distribution reliably.

Several strengths and limitations of this study should be considered. Strengths include the use of a well-characterized HCAR1 KO model, integration of behavioral and electrophysiological analyses within the same animals, and inclusion of the human hippocampal tissue from a medication-naive cohort. Limitations include that experimentally naive WT and HCAR1 KO mice were used without induction of depression. Such models may be required to reveal HCAR1-dependent behavioral effects that are not apparent under baseline physiological conditions. Another limitation is the lack of direct lactate measurements in the present cohort and the exclusion of the planned lactate-injection group. The identical treadmill protocol has previously been validated extensively in the same HCAR1 mouse line, same treadmill protocol, and same laboratory setting (Morland et al., 2017), supporting the relevance of the paradigm for lactate-associated exercise responses. However, because the lactate-injection group was excluded, the present study cannot directly determine whether the observed effects reflect receptor-independent mechanisms, insufficient HCAR1 ligand engagement, or compensatory adaptations to constitutive HCAR1 deletion. Finally, the human tissue analysis was limited in size and scope, precluding detailed assessment of cell type-specific expression patterns or subtle diagnosis-related differences.

Taken together, our findings do not support a broad requirement for HCAR1 in the behavioral measures assessed here or in the main behavioral and hippocampal adaptations observed following exercise in healthy animals. HCAR1 may contribute to more subtle, state-dependent modulation of hippocampal circuit responsiveness, as suggested by the genotype-dependent differences in DG excitability and resting corticosterone levels. In the available human cohort, HCAR1 showed a conserved perivascular distribution in the hippocampus without evidence of marked alterations in expression or perivascular localization in MDD. These findings constrain HCAR1's contribution to exercise-associated behavioral and hippocampal outcomes under physiological conditions, but do not exclude a role under pathological or high-demand conditions. Future studies incorporating disease-relevant models, direct manipulation of lactate signaling, and in vivo metabolic and endocrine measurements will be necessary to define the conditions under which HCAR1 contributes to brain function.

## Synthesis

Reviewing Editor: Adrien Peyrache, McGill University Decisions are customarily a result of the Reviewing Editor and the peer reviewers coming together and discussing their recommendations until a consensus is reached. When revisions are invited, a fact-based synthesis statement explaining their decision and outlining what is needed to prepare a revision will be listed below. The following reviewer(s) agreed to reveal their identity: NONE.

The authors have addressed all the remaining concerns and the paper is now suitable for publication.

## Author Response

We thank the Editor and Reviewers for their careful evaluation and constructive feedback. We agree with the central concern that the original manuscript overextended its conclusions regarding the role of HCAR1 in depression-related processes relative to the experimental design. In response, we have substantially revised the manuscript to better align the conceptual framing with the data.

Specifically, we have reframed the study from testing whether HCAR1 mediates antidepressant effects of exercise to a more focused investigation of whether HCAR1 is required for behavioral and hippocampal adaptations to exercise under physiological conditions, while using human postmortem hippocampal tissue to assess whether HCAR1 expression or spatial organization is altered in MDD. This change is reflected throughout all parts of the manuscript.

In addition, we have strengthened the manuscript by (i) integrating corticosterone measurements to assess stress-related confounding, (ii) refining the interpretation of behavioral and electrophysiological findings, (iii) clarifying the role of the human tissue analysis as providing anatomical and translational context rather than testing disease mechanisms, and (iv) expanding engagement with the literature linking lactate signaling to mood regulation.

Below, we address each point in detail.

1) The exercise paradigm that was chosen may not produce the intended effect. Here, forced treadmill exercise was chosen, which in some cases can be stressful and not "antidepressant" (see https://pmc.ncbi.nlm.nih.gov/articles/PMC5145912/). Indeed, the decrease in grooming time and sociability indicate that the forced exercise was stressful. It is thus difficult to distinguish antidepressant-like effects from stress-related behavioral changes. Voluntary wheel running would be a much better choice.

We agree that forced treadmill exercise can introduce a stress component and that this complicates interpretation of behavioral outcomes in the context of mood-related assays. However, to investigate lactate-induced effects of HCAR1 activation, exercise above the anaerobic threshold is required. This can only be reliably ensured by using treadmill running, which permits experimental control of both exercise intensity (by comparison to the MECT results) and duration. In contrast, wheel-running only allows assessment - rather than experimental control - of exercise intensity and duration. In the revised manuscript, we have explicitly acknowledged the possibility that forced exercise may induce stress as a limitation and adjusted the interpretation accordingly. We have now included corticosterone measurements from plasma collected approximately 4 hours after the final exercise session; similar timing to the behavioral tests. These data do not support a uniform, sustained elevation of corticosterone in exercised animals at the time of sacrifice. Instead, corticosterone levels were highly variable in WT mice and did not differ significantly between exercised and sedentary WT groups. Interestingly, sedentary HCAR1-KO mice displayed consistently elevated corticosterone levels compared to WT controls, whereas exercised KO mice showed lower and more variable levels.

These findings suggest that the exercise paradigm did not produce a persistent generalized stress response at the endpoint. We have incorporated these data into both the Results and Discussion and tempered our interpretation of the behavioral findings accordingly.

2) The absence of a baseline depressive or anxiety-like phenotype in HCAR1 knockout mice severely limits the interpretability of the behavioral findings. The splash test and three-chamber sociability task are unlikely to detect subtle mood-related alterations in healthy animals, and without a chronic stress or other validated depression model, the data cannot meaningfully address the role of HCAR1 in depression-related behavior.

We agree that the absence of a robust baseline behavioral phenotype limits the ability to interpret the data in the context of depression models. In the revised manuscript, we have explicitly reframed the study to avoid making claims about depression mechanisms.

Rather than interpreting the findings as evidence for or against antidepressant effects, we now present the data as demonstrating that HCAR1 is not required for baseline behavioral alterations or for the primary behavioral adaptations to exercise in healthy animals.

We also emphasize that the behavioral assays used provide indirect measures of affective function and that more disease-relevant models (e.g., chronic stress paradigms) will be necessary to determine whether HCAR1 contributes to mood-related processes under pathological conditions.

3) Mechanistic insight is further limited by the absence of direct lactate manipulation and by the lack of information on brain lactate concentrations in response to the exercise paradigm used. The presented logic indicates that exercise increases circulating lactate, that then activates HCAR1 in the brain. Lactate was not measured in either brain or blood. Without a lactate injection making it impossible to know that exercise produced the intended effect. It thus makes it unclear whether the largely negative findings reflect receptor independence or insufficient ligand engagement. Cortisol should also be measured to ensure that the forced exercise was not stressful.

We acknowledge this limitation and have clarified it more explicitly in the revised manuscript. While we did not measure lactate levels directly, the treadmill protocol used has previously been shown to robustly elevate circulating lactate and induce HCAR1-dependent effects in the brain in this mouse model (Morland et al., 2017), which we now state more clearly.

Importantly, we have revised the conceptual framing of the study to reflect that we are testing the necessity of HCAR1 for exercise-induced adaptations rather than directly interrogating lactate signaling mechanisms. Under this framework, the observation that behavioral and electrophysiological responses to exercise are largely preserved in HCAR1-KO mice suggests that these adaptations can occur independently of HCAR1 signaling.

We agree that direct lactate measurements or pharmacological manipulation would provide additional mechanistic insight, and we now highlight this as an important direction for future studies.

4) The electrophysiological analyses identify statistically significant but subtle differences in dentate gyrus responsiveness whose functional relevance is unclear. These results remain largely descriptive and do not substantially advance understanding of HCAR1 function in mood-related circuits.

We agree that the electrophysiological differences observed are modest. In the revised manuscript, we have refined the interpretation of these findings and reduced speculative mechanistic explanations.

We now describe these results as indicating a subtle modulation of dentate gyrus input-output coupling by HCAR1 in response to exercise, rather than broad alterations in synaptic plasticity. We have removed speculation regarding neurogenesis and clarified that hippocampal neurogenesis appears to be unaffected by HCAR1 deletion based on previous studies.

These changes place the electrophysiological findings in the context of circuit-level modulation rather than a primary mechanism of behavioral change.

5) The human immunohistochemistry analysis is underpowered, limiting conclusions regarding diagnosis-specific differences in HCAR1 expression or localization.

We agree that the human cohort is limited in size and does not allow detection of subtle or cell type-specific differences. In the revised manuscript, we clarify that the primary purpose of this analysis is to characterize the spatial distribution of HCAR1 in the human hippocampus and to assess whether large-scale differences in expression are present in MDD. We also note that access to well-characterized, medication-naive human hippocampal tissue is inherently limited, making large cohorts difficult to obtain, and that studies of this type are therefore primarily suited to detecting robust, large-scale alterations rather than subtle effects. Accordingly, we have removed any implication that these data demonstrate disease-specific mechanisms and now explicitly state that the absence of significant differences does not exclude more subtle alterations.

6) Finally, the manuscript also does not sufficiently engage with the literature linking lactate to antidepressant effects and exercise-induced mood regulation.

We agree with this point and have substantially revised both the Introduction and Discussion to better engage with the literature on lactate signaling in mood regulation and exercise-induced brain adaptations. In the revised manuscript, we now explicitly incorporate evidence showing that lactate can influence neuronal activity, promote stress resilience, and produce antidepressant-like behavioral effects, as well as studies linking exercise-induced lactate to hippocampal plasticity.

Importantly, we have also clarified how our findings relate to this body of work. Rather than challenging the broader role of lactate in mood-related processes, our data indicate that HCAR1 is not required for the behavioral and physiological adaptations observed under the present experimental conditions. This suggests that lactate's effects are not exclusively mediated through HCAR1 and may instead involve multiple signaling and metabolic pathways or depend on specific physiological or pathological contexts. With these revisions, our findings refine the interpretation of the current models, rather than contradicting the existing literature.

## References

[B1] Ahmed K, Tunaru S, Tang C, Müller M, Gille A, Sassmann A, Hanson J, Offermanns S (2010) An autocrine lactate loop mediates insulin-dependent inhibition of lipolysis through GPR81. Cell Metab 11:311–319. 10.1016/j.cmet.2010.02.012 20374963

[B2] Boldrini M, Hen R, Underwood MD, Rosoklija GB, Dwork AJ, Mann JJ, Arango V (2012) Hippocampal angiogenesis and progenitor cell proliferation are increased with antidepressant use in major depression. Biol Psychiatry 72:562–571. 10.1016/j.biopsych.2012.04.024 22652019 PMC3438317

[B3] Briquet M, Rocher A-B, Alessandri M, Rosenberg N, de Castro Abrantes H, Wellbourne-Wood J, Schmuziger C, Ginet V, Puyal J, Pralong E (2022) Activation of lactate receptor HCAR1 down-modulates neuronal activity in rodent and human brain tissue. J Cereb Blood Flow Metab 42:1650–1665. 10.1177/0271678X221080324 35240875 PMC9441721

[B4] Carrard A, Elsayed M, Margineanu M, Boury-Jamot B, Fragnière L, Meylan E, Petit J, Fiumelli H, Magistretti PJ, Martin J (2018) Peripheral administration of lactate produces antidepressant-like effects. Mol Psychiatry 23:392–399. 10.1038/mp.2016.179 27752076 PMC5794893

[B5] Carrard A, Cassé F, Carron C, Burlet-Godinot S, Toni N, Magistretti PJ, Martin J-L (2021) Role of adult hippocampal neurogenesis in the antidepressant actions of lactate. Mol Psychiatry 26:6723–6735. 10.1038/s41380-021-01122-0 33990772 PMC8760055

[B6] Chen F, Bertelsen AB, Holm IE, Nyengaard JR, Rosenberg R, Dorph-Petersen K-A (2020) Hippocampal volume and cell number in depression, schizophrenia, and suicide subjects. Brain Res 1727:146546. 10.1016/j.brainres.2019.146546 31715144

[B7] Czéh B, Lucassen PJ (2007) What causes the hippocampal volume decrease in depression? Are neurogenesis, glial changes and apoptosis implicated? Eur Arch Psychiatry Clin Neurosci 257:250–260. 10.1007/s00406-007-0728-0 17401728

[B8] de Castro Abrantes H, Briquet M, Schmuziger C, Restivo L, Puyal J, Rosenberg N, Rocher A-B, Offermanns S, Chatton J-Y (2019) The lactate receptor HCAR1 modulates neuronal network activity through the activation of Gα and Gβγ subunits. J Neurosci 39:4422–4433. 10.1523/JNEUROSCI.2092-18.2019 30926749 PMC6554634

[B9] El Hayek L, et al. (2019) Lactate mediates the effects of exercise on learning and memory through SIRT1-dependent activation of hippocampal brain-derived neurotrophic factor (BDNF). J Neurosci 39:2369–2382. 10.1523/jneurosci.1661-18.2019 30692222 PMC6435829

[B10] Fuss J, Ben Abdallah NMB, Vogt MA, Touma C, Pacifici PG, Palme R, Witzemann V, Hellweg R, Gass P (2010) Voluntary exercise induces anxiety-like behavior in adult C57BL/6J mice correlating with hippocampal neurogenesis. Hippocampus 20:364–376. 10.1002/hipo.20634 19452518

[B11] Hadzic A, Nguyen TD, Hosoyamada M, Tomioka NH, Bergersen LH, Storm-Mathisen J, Morland C (2020) The lactate receptor HCA(1) is present in the choroid plexus, the tela choroidea, and the neuroepithelial lining of the dorsal part of the third ventricle. Int J Mol Sci 21:6457. 10.3390/ijms21186457 32899645 PMC7554735

[B12] Herrera-López G, Griego E, Galván EJ (2020) Lactate induces synapse-specific potentiation on CA3 pyramidal cells of rat hippocampus. PLoS One 15:e0242309. 10.1371/journal.pone.0242309 33180836 PMC7660554

[B13] Hu J, Cai M, Shang Q, Li Z, Feng Y, Liu B, Xue X, Lou S (2021) Elevated lactate by high-intensity interval training regulates the hippocampal BDNF expression and the mitochondrial quality control system. Front Physiol 12:629914. 10.3389/fphys.2021.629914 33716776 PMC7946986

[B14] Hwang D, Kim J, Kyun S, Jang I, Kim T, Park H-Y, Lim K (2023) Exogenous lactate augments exercise-induced improvement in memory but not in hippocampal neurogenesis. Sci Rep 13:5838. 10.1038/s41598-023-33017-1 37037890 PMC10086059

[B15] Karnib N, et al. (2019) Lactate is an antidepressant that mediates resilience to stress by modulating the hippocampal levels and activity of histone deacetylases. Neuropsychopharmacology 44:1152–1162. 10.1038/s41386-019-0313-z 30647450 PMC6461925

[B16] Kennedy L, Glesaaen ER, Palibrk V, Pannone M, Wang W, Al-Jabri A, Suganthan R, Meyer N, Austbø ML, Lin X (2022) Lactate receptor HCAR1 regulates neurogenesis and microglia activation after neonatal hypoxia-ischemia. eLife 11:e76451. 10.7554/eLife.76451 35942676 PMC9363115

[B17] Lambertus M, Øverberg LT, Andersson KA, Hjelden MS, Hadzic A, Haugen ØP, Storm-Mathisen J, Bergersen LH, Geiseler S, Morland C (2021) L-lactate induces neurogenesis in the mouse ventricular-subventricular zone via the lactate receptor HCA1. Acta Physiol 231:e13587. 10.1111/apha.13587 33244894

[B18] Lambertus M, Geiseler S, Morland C (2024) High-intensity interval exercise is more efficient than medium intensity exercise at inducing neurogenesis. J Physiol 602:7027–7042. 10.1113/JP287328 39580614 PMC11649522

[B19] Lauritzen KH, Morland C, Puchades M, Holm-Hansen S, Hagelin EM, Lauritzen F, Attramadal H, Storm-Mathisen J, Gjedde A, Bergersen LH (2014) Lactate receptor sites link neurotransmission, neurovascular coupling, and brain energy metabolism. Cereb Cortex 24:2784–2795. 10.1093/cercor/bht136 23696276

[B20] Morland C, Lauritzen KH, Puchades M, Holm-Hansen S, Andersson K, Gjedde A, Attramadal H, Storm-Mathisen J, Bergersen LH (2015) The lactate receptor, G-protein-coupled receptor 81/hydroxycarboxylic acid receptor 1: expression and action in brain. J Neurosci Res 93:1045–1055. 10.1002/jnr.23593 25881750

[B21] Morland C, Andersson KA, Haugen ØP, Hadzic A, Kleppa L, Gille A, Rinholm JE, Palibrk V, Diget EH, Kennedy LH (2017) Exercise induces cerebral VEGF and angiogenesis via the lactate receptor HCAR1. Nat Commun 8:15557. 10.1038/ncomms15557 28534495 PMC5457513

[B22] Müller P, Duderstadt Y, Lessmann V, Müller NG (2020) Lactate and BDNF: key mediators of exercise induced neuroplasticity? J Clin Med 9:1136. 10.3390/jcm9041136 32326586 PMC7230639

[B23] Nezhady MAM, Cagnone G, Joyal J-S, Chemtob S (2023) Lack of HCAR1, the lactate GPCR, signaling promotes autistic-like behavior. Cell Commun Signal 21:196. 10.1186/s12964-023-01188-z 37940970 PMC10634184

[B24] Pollak DD, Rey CE, Monje FJ (2010) Rodent models in depression research: classical strategies and new directions. Ann Med 42:252–264. 10.3109/07853891003769957 20367120

[B25] Ross RE, VanDerwerker CJ, Saladin ME, Gregory CM (2023) The role of exercise in the treatment of depression: biological underpinnings and clinical outcomes. Mol Psychiatry 28:298–328. 10.1038/s41380-022-01819-w 36253441 PMC9969795

[B26] Schuch FB, Deslandes AC, Stubbs B, Gosmann NP, da Silva CTB, de Almeida Fleck MP (2016) Neurobiological effects of exercise on major depressive disorder: a systematic review. Neurosci Biobehav Rev 61:1–11. 10.1016/j.neubiorev.2015.11.012 26657969

[B27] Van Praag H, Kempermann G, Gage FH (1999) Running increases cell proliferation and neurogenesis in the adult mouse dentate gyrus. Nat Neurosci 2:266–270. 10.1038/6368 10195220

[B28] Zhang S, Xia J, He W, Zou Y, Liu W, Li L, Huang Z, Li Q, Qi Z, Liu W (2025) From energy metabolism to mood regulation: the rise of lactate as a therapeutic target. J Adv Res 80:535–554. 10.1016/j.jare.2025.04.018 40262720 PMC12869207

